# Stokes and Bell’s Practice

**Published:** 1841-01

**Authors:** 


### STOKES AND BELL'S PRACTICE.

We have received a copy of this work from the publishers, and shall take the earliest occasion to give our readers our opinion of its merits. We have had leisure merely to look into one or two of the lectures of Dr. Bell, but from that slight inspection we are prepared to find his additions to the labors of Dr. Stokes highly valuable. No writer on practical medicine of the present day has higher claims to the confidence of the profession, than the distinguished teacher at Dublin; and Dr. Bell has long stood in the first rank of American medical writers. Besides his lectures, twelve in number, Dr. Bell has added copious notes to the lectures of Dr. Stokes. The lectures are devoted to typhus fever; congestive fever, its pathology and treatment, at great but not unnecessary length; rheumatism; and laryngitis. The use of sulphate of quinine in large doses—a practice introduced by physicians of the south-west, is strongly commended by Dr. Bell. His analysis of the operation of calomel is clear and able.

The volume contains 672 8vo. pages, and is handsomely printed on good paper.

